# Optimizing blood management in burn surgery: a meta-analysis of tranexamic acid vs. placebo

**DOI:** 10.1186/s12893-025-03014-4

**Published:** 2025-07-11

**Authors:** Mohamed Abdo Khalafallah, Jana Ahmed Elsead, Fatima Azzam Aboud, Basmala Mohamed Koraim, Islam Saeed Elhois

**Affiliations:** 1 Faculty of Medicine, Alexandria University, Alexandria, Egypt; 2 Faculty of Medicine, Cairo University, Cairo, Egypt; 3 Qena General Hospital, Ministry of Health and Population, Qena, Egypt

**Keywords:** Tranexamic acid, TXA, Burn surgery, Blood loss, Blood transfusion.

## Abstract

**Background:**

Early debridement and grafting improve burn outcomes but pose significant blood loss risks. Tranexamic acid (TXA), an antifibrinolytic agent, reduces hemorrhage without increasing Venous thromboembolism (VTE) risk. While widely used in other surgeries, its role in burn surgery remains unclear. This meta-analysis evaluates TXA’s efficacy in improving surgical outcomes in burn patients.

**Methods:**

We searched PubMed, Scopus, Web of Science, Cochrane, and Springer databases (last search: February 2025). Eligible RCTs compared TXA vs. placebo in burn surgery. Primary outcomes included blood loss (ml), transfusion need, hemoglobin change (g/dL), and hematocrit change (%). We conducted sensitivity, cumulative, and meta-regression analysis for all outcomes and Grading of Recommendations Assessment, Development, and Evaluation (GRADE) for primary outcomes.

**Results:**

We included five studies containing 227 patients. TXA significantly reduced operative blood loss (MD: -181.52 mL; *p* = 0.00; moderate certainty; I² = 61.46%) and transfusion need (RR: 0.52; *p* = 0.01; moderate certainty; I² = 0%). However, TXA did not significantly affect changes in hemoglobin (MD: 0.06; *p* = 0.94; low certainty; I² = 91.29%) or hematocrit levels (MD: 0.19; *p* = 0.90; very low certainty; I² = 88.94%).

**Conclusion:**

TXA significantly reduces total operative blood loss and transfusion needs with moderate certainty. However, it does not significantly impact hemoglobin or hematocrit levels. Secondary outcomes showed no significant differences, including operative time, hospitalization length, and infection rates.

**Trial registration:**

Not applicable.

**Supplementary Information:**

The online version contains supplementary material available at 10.1186/s12893-025-03014-4.

## Introduction

Early debridement and grafting remain the gold standard for managing burn patients, as this approach has been shown to enhance graft success rates, reduce the risk of infections, and mitigate the likelihood of sepsis and multi-organ failure [1, 2].

However, surgical debridement in burn patients is associated with significant blood loss, posing a major challenge and serving as an important predictor of mortality [3]. To address this concern, surgeons have employed various hemostatic strategies, including the use of topical adrenaline, tourniquets, and tranexamic acid (TXA) to minimize intraoperative blood loss [4].

TXA, a synthetic lysine analogue with antifibrinolytic properties, functions by inhibiting the conversion of plasminogen to plasmin, thereby preventing fibrin degradation and stabilizing clot formation [5]. Notably, while TXA exerts a pro-coagulant effect, current evidence suggests that it does not increase the incidence of venous thromboembolism (VTE) [6]. However, some concerns have been raised about its potential to increase VTE risk in severe burn patients due to the hypercoagulable state associated with burns [7, 8]. Notably, existing evidence generally suggests TXA does not elevate VTE incidence in other clinical contexts, but this specific concern in burn populations requires further scrutiny.

Nevertheless, TXA’s ability to reduce perioperative blood loss may contribute to a decreased need for transfusions, ultimately lowering the risk of infection and mortality in burn patients. Its efficacy in reducing hemorrhagic complications has been well-established in other surgical specialities, including cardiac, orthopaedic, and obstetric surgery [9–11].

Despite these promising theoretical benefits, the literature on TXA use in burn surgery remains limited. Therefore, we conducted a systematic review and meta-analysis to assess whether TXA can significantly improve surgical outcomes in burn patients.

## Methods

This review was performed according to the guidelines of the Preferred Reporting Items for Systematic Reviews and Meta-Analyses (PRISMA 2020) [12]. Furthermore, we registered the study protocol in PROSPERO with registration number CRD42025645808.

### Eligibility criteria

Studies were selected based on predefined inclusion and exclusion criteria.

- **Inclusion criteria** required that studies be randomized controlled trials (RCTs) comparing the effectiveness of TXA to placebo (saline) in patients undergoing burn surgery. Burn surgery was defined as surgical interventions for thermal injury, including tangential excision of necrotic tissue, fascial excision, and skin grafting. Studies were included if they explicitly described the surgical procedure. Only studies published in the English language were considered.
- **Exclusion criteria** encompassed study protocols, non-randomized controlled trials, reviews, meta-analyses, letters, book chapters, and conference abstracts. Additionally, studies conducted on non-human subjects were excluded.

### Literature search strategy

We searched PubMed, Scopus, Web of Science, Cochrane Library and Springer electronic databases. The authors conducted the last search in February 2025. We used a specific search strategy for each database. Furthermore, the search strategies used in the screening process were presented in Supplementary File 1. Table 1. The authors also checked the reference lists of relevant articles to ensure no studies were missing. Duplicates were identified and removed during the initial screening process.

### Selection of studies

Two independent reviewers screened title and abstract of the studies according to the eligibility criteria, and the studies that met the criteria, entered full text screening. Any conflicts between reviewers were resolved by discussion or by involving third reviewer.

### Data extraction

Data were extracted using a Microsoft Excel spreadsheet to collect relevant variables systematically. The extracted data included key study parameters such as inclusion and exclusion criteria, details of the intervention and control groups, primary and secondary outcomes, total sample size, and follow-up duration.

Additionally, demographic characteristics were recorded, including mean age, gender distribution, body mass index (BMI), total body surface area (TBSA) affected, burn degree, presence of inhalation injury, and initial hematocrit and hemoglobin levels (mean ± SD).

Two independent authors reviewed the extracted data to ensure accuracy and consistency. Any discrepancies were resolved through discussion, and if necessary, a third reviewer was consulted to reach a consensus. When the original authors did not report baseline characteristics, we coded these as missing (N/A).

### Quality assessment

The risk of bias 2.0 (RoB 2) tool [13] is a Cochran tool used to assess the quality of the included studies according to main domains such as Random sequence generation, Allocation concealment, Blinding of participants and employs, Blinding of outcome assessment, incomplete outcome data, Selective reporting, and other factors. Two independent reviewers assessed the quality, and any conflicts were resolved by discussion or involving a third reviewer.

### Outcomes measurement

- The primary outcomes include total operative-related blood loss (mL), the need for transfusion, the difference in hemoglobin levels (g/dL), and the differences in hematocrit levels (%).
- The secondary outcomes consist of operative time (minutes), length of hospitalization (days), total units of packed red blood cells (PRBC) transfused, the presence of post-operative infections, and the amounts of intra-operative crystalloids and colloids used (units).
- The term ‘need for transfusion’ indicates whether patients received any transfusion, reflecting a binary outcome. In contrast, ‘units of PRBC transfused’ captures the amount of blood given to those who were transfused, reflecting a continuous outcome.

### Data synthesis and statistical analysis

We performed all statistical analyses using STATA 17 software, with a p-value < 0.05 considered statistically significant. We analyzed continuous outcomes using the mean difference (MD) with 95% confidence intervals (CIs) to estimate effect sizes. We calculated the risk ratio (RR) with 95% CIs for dichotomous outcomes. We assessed heterogeneity across studies using the I² statistic, where an I² value of 0% indicates no observed heterogeneity, while values exceeding 50% suggest substantial heterogeneity. We employed a random-effects model to account for heterogeneity and to generalize findings across diverse study populations.

We performed meta-regression analysis to explore the potential moderating effects of age and sex (specifically male) on the outcomes. This analysis helped identify whether these variables significantly influenced the effect sizes observed in the studies. We selected age and sex (proportion male) for meta‑regression because these demographic variables were consistently and comprehensively reported across all included trials, allowing for adequate power and comparability. Other potential moderators—TBSA burned, burn depth and TXA timing of administration—were considered a priori but could not be tested formally due to incomplete reporting or insufficient between‑study variability.

We conducted sensitivity analysis to assess the robustness of the meta-analysis findings. This involved re-calculating the overall effect sizes after excluding individual studies to determine if any single study influenced the results.

Cumulative analysis examined how the effect sizes evolved as new studies were added. This analysis provided insights into the stability of the findings and the impact of newer studies on the overall results.

We used the Grading of Recommendations Assessment, Development, and Evaluation (GRADE) approach in similar surgical contexts, such as Wang et al.’s (2024) [14] to assess the certainty of evidence. This method evaluates the quality of evidence based on factors such as risk of bias, inconsistency, indirectness, imprecision, and publication bias [15]. While using GRADE considers publication bias, due to the limited number of included studies, publication bias could not be assessed using Egger’s test recommendations or funnel plot visualization [16]. Certainty levels were classified in the following manner: moderate certainty indicated that further research might alter the effect estimate, low certainty suggested high uncertainty with potential for significant changes, and very low certainty implied that the actual effect could differ substantially from the observed estimate [17].

## Results

### Study selection

**Fig. 1:**
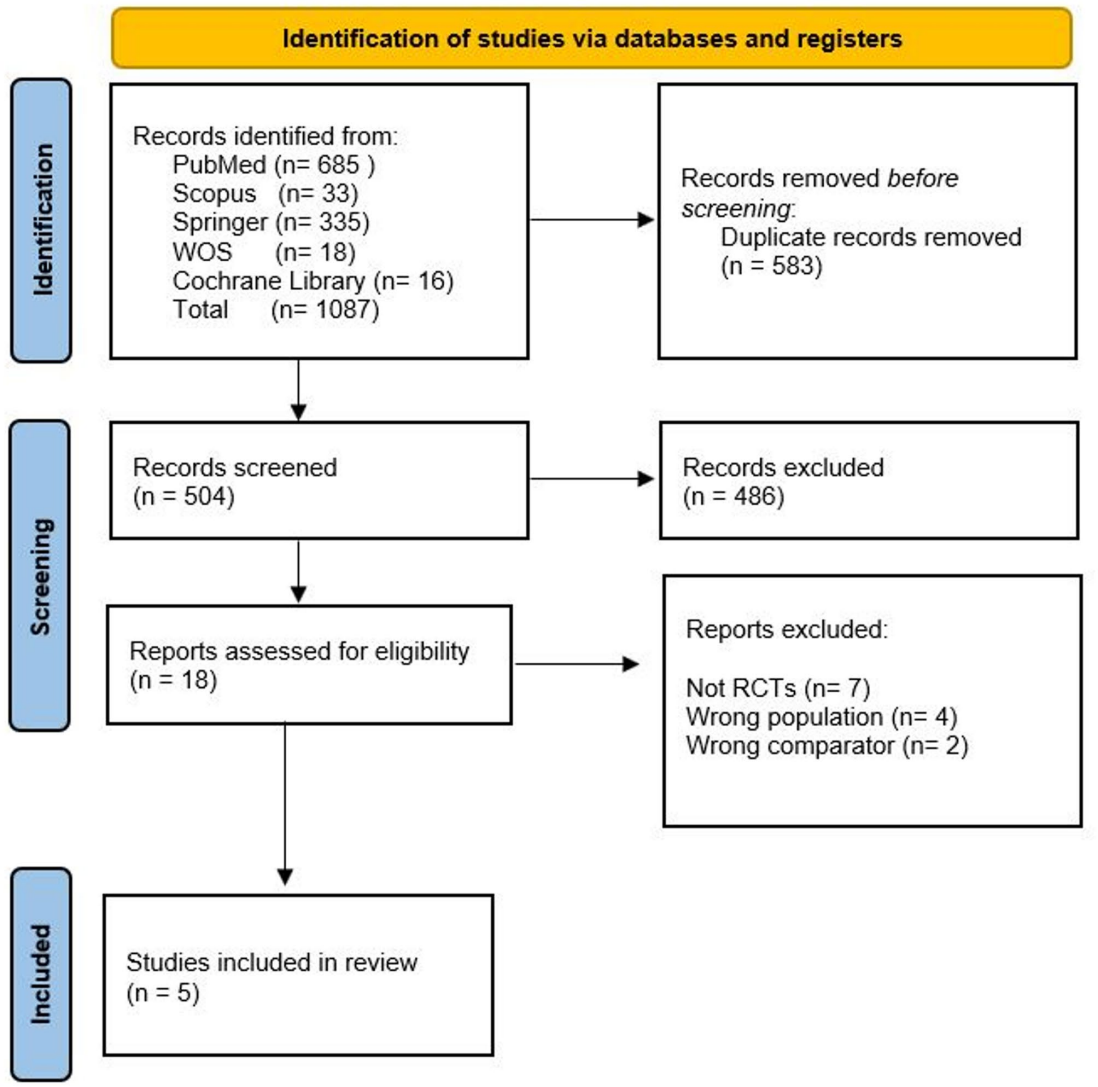
Prisma flow diagram

The systematic review followed a structured screening process based on PRISMA guidelines. Initially, 1,087 records were retrieved from multiple databases, including PubMed (*n* = 685), Scopus (*n* = 33), Springer (*n* = 335), Web of Science (*n* = 18), and the Cochrane Library (*n* = 16). After removing 583 duplicates, 504 unique records remained for screening. Following title and abstract screening, 486 studies were excluded due to irrelevance. The remaining 18 full-text articles were assessed for eligibility, excluding seven non-RCTs, four studies with the wrong population, and two with an inappropriate comparator. Five studies were included in this review, with a combined sample size of 227 participants. This rigorous selection process is presented in Fig. 1.

The studies were conducted across diverse geographical locations, including India, Norway, Mexico, and Iran, reflecting a broad spectrum of clinical settings. The intervention groups received varied doses of intravenous TXA, ranging from 10 mg/kg to 15 mg/kg, administered over specific durations and diluted in saline as described. Control groups were generally treated with saline solutions as comparators. Follow-up durations varied among studies, with some assessing outcomes immediately and at 24 h post-operation, while others extended follow-up to 3 months or beyond. A summary of the common characteristics of the included studies is presented in Table 1, and baseline characteristics are presented in Supplementary File 1, Table 2.

**Table 1:** Summary of the included studies

Study ID	Country	Inclusion Criteria	Exclusion Criteria	Intervention	Control	Primary Outcomes	Secondary Outcomes	Sample Size)	Follow-up
Ajai 2022	India	Patients aged 15–55 years with deep dermal thermal burns < 30% TBSA undergoing tangential excision	Patients with third-degree burns, chemical/electrical burns, head injury, blunt trauma, renal dysfunction, coagulopathy, bleeding disorders, hypercoagulability, thromboembolic history, pregnancy, transfusion refusal, or TXA hypersensitivity.	TXA 15 mg/kg IV, diluted in 10 ml, infused over 15 min, 10 min pre-surgery.	10 ml saline IV, administered similarly.	Blood loss /burn area excised (ml/cm2)	Total blood loss (ml) Intraoperative crystalloids (units) Intraoperative colloids (units) Postoperative hemoglobin (24 h) (g/dL) Total PRBC transfused (units) Hospitalization length (days) Graft take (%)	30	3 months
Bhatia 2017	India	Patients aged 18–50 years (ASA I/II) with > 20% TBSA third-degree burns, scheduled for debridement/eschar removal ± skin grafting under general anesthesia ≥ 10 days post-burn.	Patients with infarction history, unstable angina, renal/hepatic insufficiency, pregnancy, ocular pathology, coagulopathy, or TXA allergy.	TXA 15 mg/kg IV, diluted to 25 ml with saline, infused over 10 min.	25 ml isotonic saline	Total amount of intraoperative blood loss (ml)	Blood transfusion requirements (trigger: Hb ≤ 7 g/dL) Hemoglobin & hematocrit changes post debridement/eschar removal & graft harvesting	50	immediate & 24 h post-operation
Colclough 2024	Norway	Patients > 18 years with burn injuries requiring STSG from ≥ 2 donor sites.	TXA allergy, pregnancy, or breastfeeding.	Topical TXA (25 mg/mL) + Epinephrine (5 µg/mL)	Topical Saline + Epinephrine (5 µg/mL)	Postoperative bleeding, measured by net dressing weight gain per wound area	Blood stain to wound area ratio. Visual comparison of the amount of blood between paired dressings. All variables were recorded on the first postoperative day. Time to re-epithelialization, defined as no oozing in the dressings. Occurrence of complications, such as: Wound infections. Thromboembolic events.	23	4 − 3 weeks
Castillo-Cardiel 2024	Mexico	First-time surgical debridement for 18–40-year-old burn patients with ≥ 20% TBSA burns (any sex).	Previous debridement, kidney disease, or drug hypersensitivity.	IV TXA solution (10 mg/kg)	10 ml saline	Intraoperative blood loss Transfusion requirement	Preoperative vs. postoperative hemoglobin & hematocrit difference	30	immediate & 24 h post-operation
Naderi 2024	iran	Patients 18–50 years with severe burns (> 20% TBSA) requiring first surgical intervention.	Patients with cardiovascular, hepatic, or renal disorders, pregnancy/lactation, ocular conditions, coagulopathies (abnormal INR), or TXA allergy.	TXA group received 10 mg/kg IV, infused in saline over 5 min.	saline	Total intraoperative blood loss, calculated using Herndon et al.’s formula: EBV × blood loss fraction (pre/post-op hematocrit) + transfusion volume.	Transfusion necessity & frequency Transfusion reactions Graft survival Operation duration (OR time) IV fluid requirements Hospital length of stay (LOS)	94	N/A

### Quality assessment

**Fig. 2:**
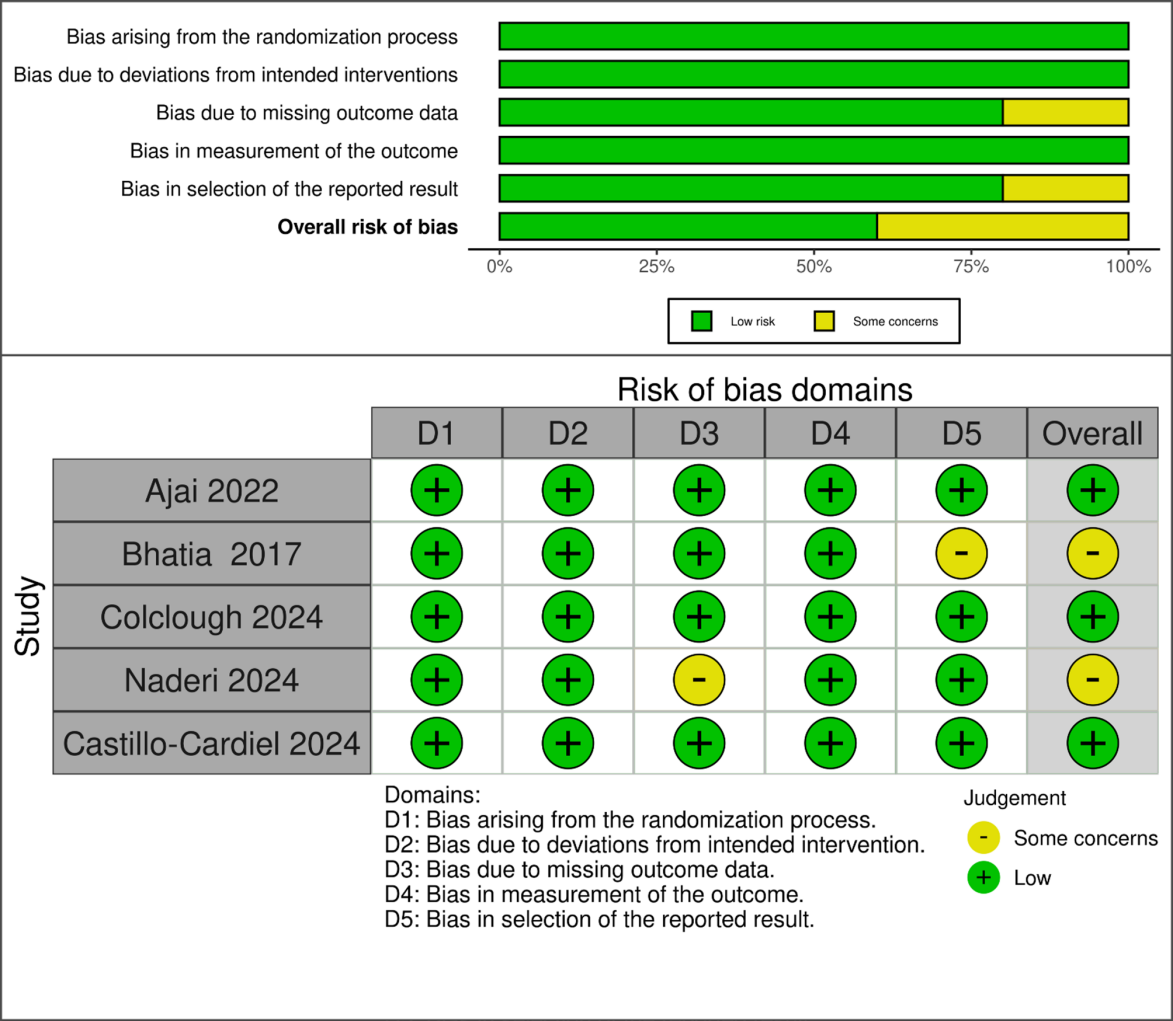
Risk of bias assessment (RoB 2)

All studies demonstrated a low risk of bias in randomization, adherence to the intended intervention, and outcome measurement, suggesting robustness in these domains. However, concerns regarding selective reporting were identified in one study (*Bhatia et al. 2017* [18]). In contrast, missing outcome data posed a potential risk in one study (*Naderi et al. 2024* [19]), which may impact result validity. Overall, three studies were classified as having a low risk of bias [20–22], whereas two studies were categorized as having some concerns regarding the risk of bias [18, 19]. Figure 2.

### Results of the Meta-Analysis

#### Primary outcomes

**Fig. 3:**
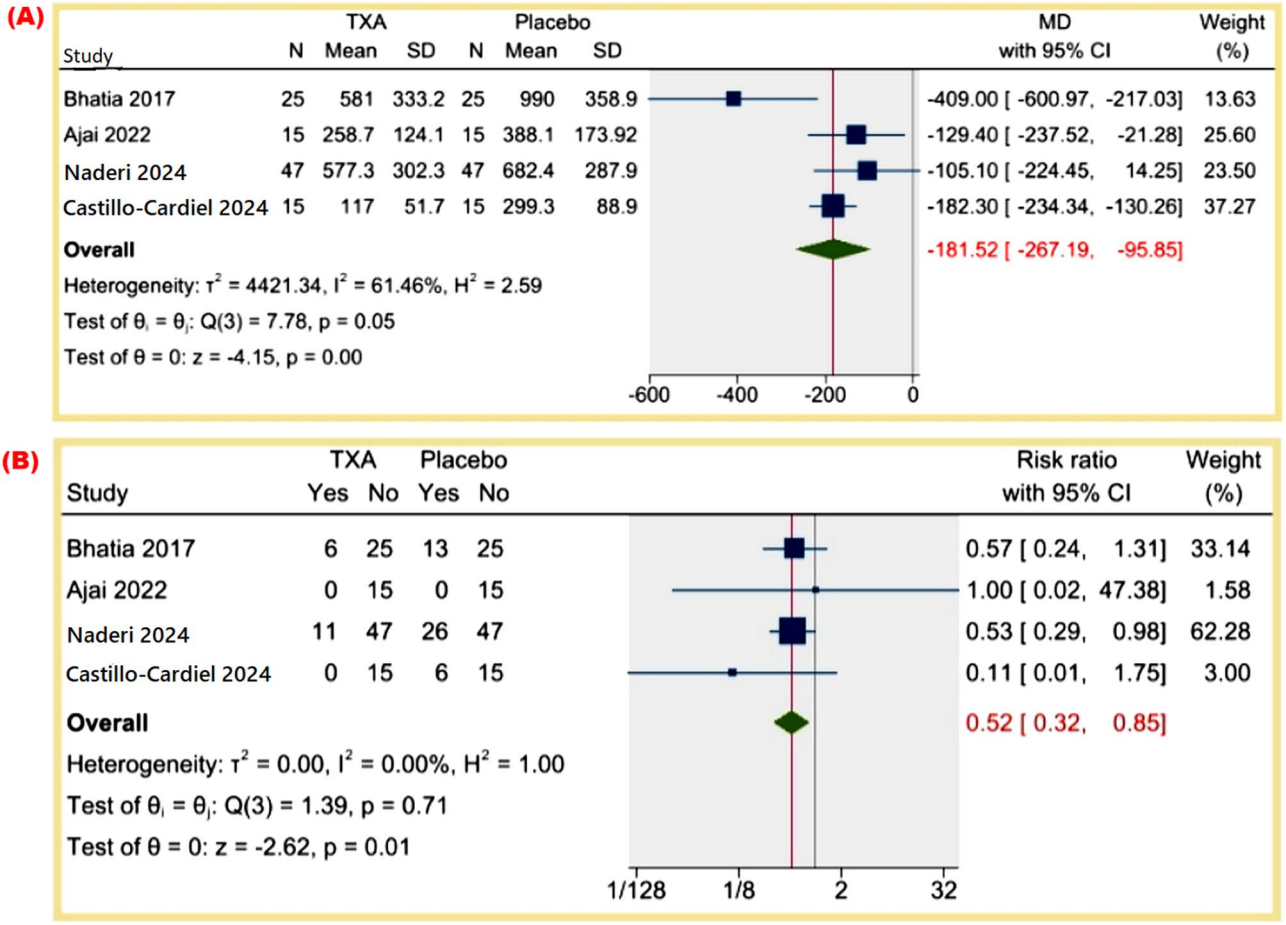
(**A**) Total operative-related blood loss (mL), (**B**) Patients Need for Transfusion

**Total operative-related blood loss (mL)**: The MD was − 181.52 mL (95% CI: -267.19 to -95.85, *p* = 0.00), indicating a statistically significant reduction in blood loss. Heterogeneity was moderate (I² = 61.46%). Figure 3A.

**Patients’ need for transfusion**: The RR was 0.52 (95% CI: 0.32 to 0.85, *p* = 0.01), demonstrating a statistically significant reduction in the need for transfusion, with no heterogeneity (I² = 0%). Figure 3B.

**Fig. 4:**
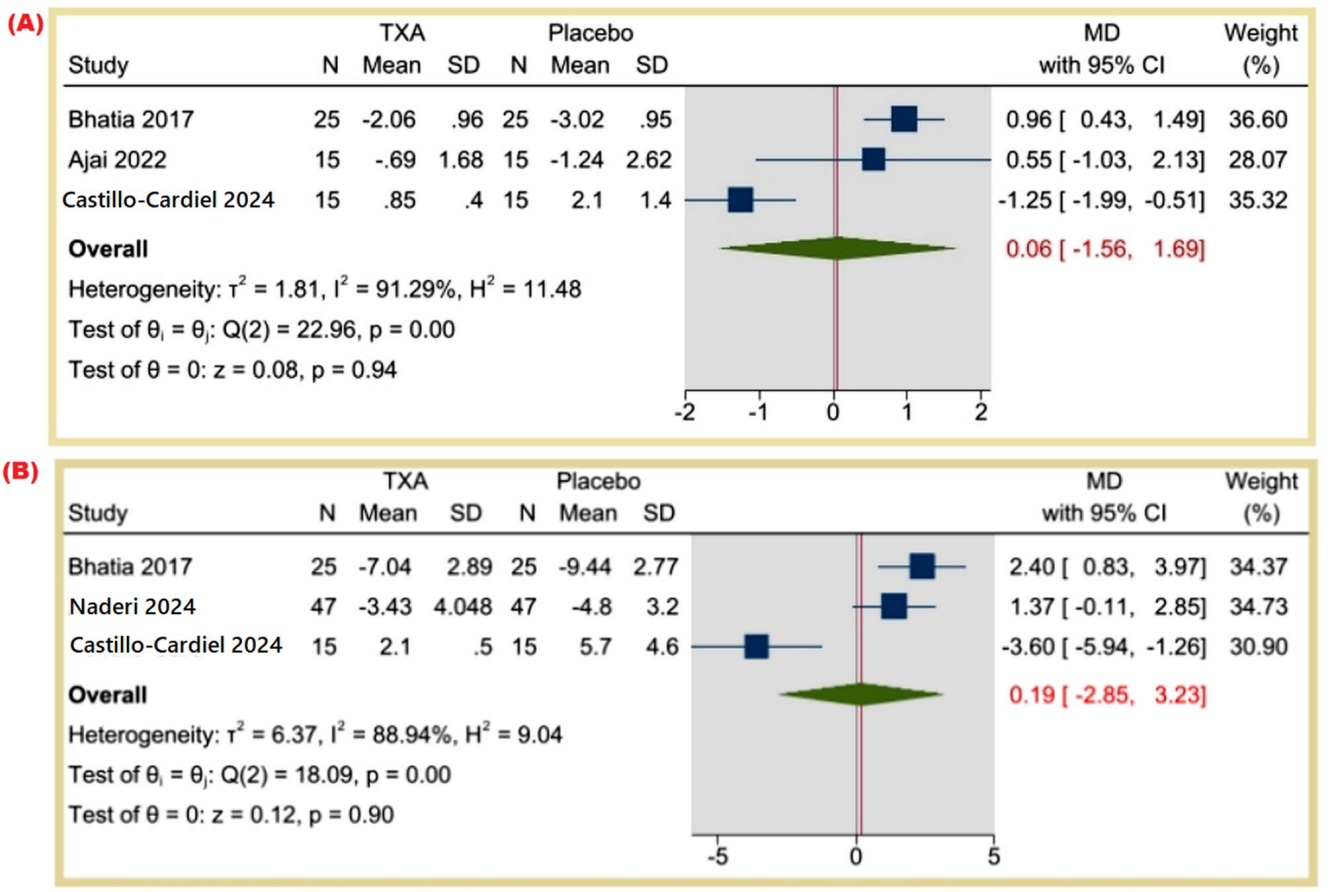
(**A**) Difference in hemoglobin levels (gr/dL), (**B**) Differences in hematocrit level (%)

**Difference in hemoglobin levels (g/dL)**: The MD was 0.06 (95% CI: -1.56 to 1.69, *p* = 0.94), suggesting no significant change in hemoglobin levels (I² = 91.29%). Figure 4A.

**Differences in hematocrit levels (%)**: The MD was 0.19 (95% CI: -2.85 to 3.23, *p* = 0.90), showing no statistically significant difference (I² = 88.94%). Figure 4B.

#### Secondary outcomes

**Fig. 5:**
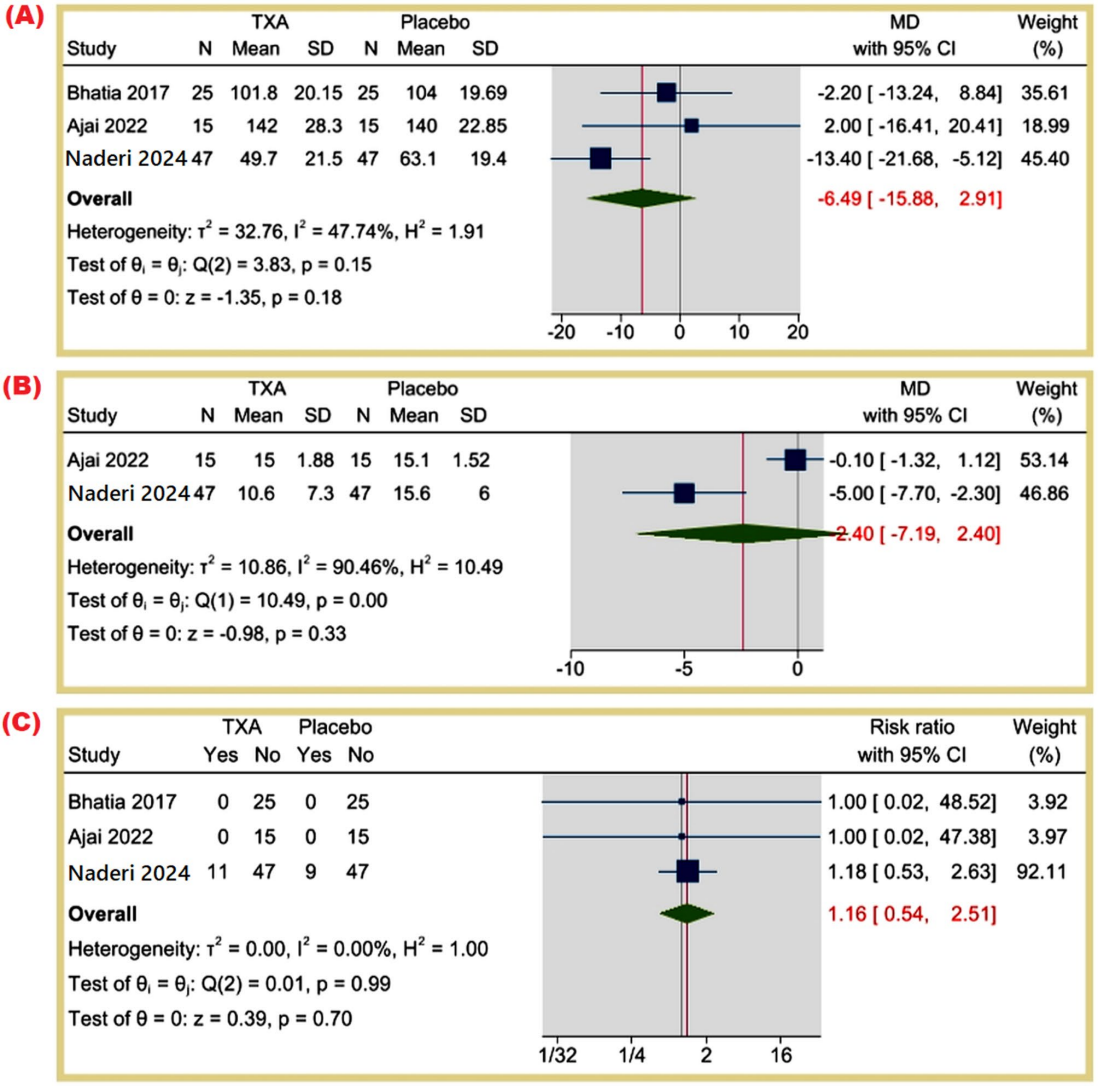
(**A**) Operative time (minutes), (**B**) Length of hospitalization (days), (**C**) Presence of post-operative infection (%)

**Operative time (minutes)**: The MD was − 6.49 min (95% CI: -15.88 to 2.91, *p* = 0.18), indicating no significant difference in operative time (I² = 47.74%). Figure 5A.

**Length of hospitalization (days)**: The MD was 2.40 days (95% CI: -7.19 to 2.40, *p* = 0.33), with high heterogeneity (I² = 90.46%). Figure 5B.

**Presence of post-operative infection**: The RR was 1.16 (95% CI: 0.54 to 2.51, *p* = 0.70), indicating no statistically significant difference, with no heterogeneity (I² = 0%). Figure 5C.

**Fig. 6:**
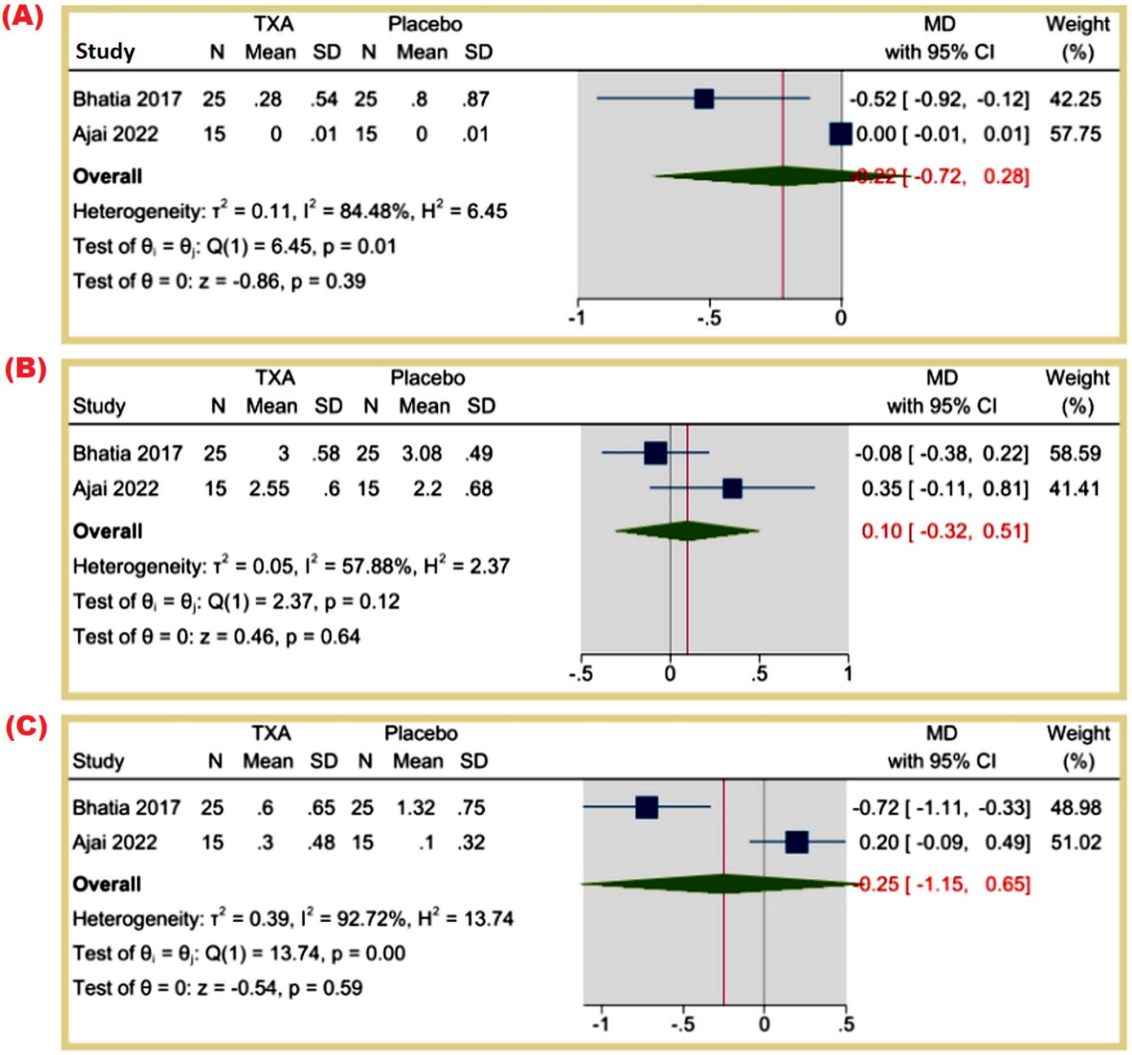
(**A**) Total PRBC transfused (units), (**B**) Intra-operative Crystalloids (units), (**C**) Intra-operative Colloids (units)

**Total PRBC transfused (units)**: The MD was − 0.22 (95% CI: -0.72 to 0.28, *p* = 0.39), suggesting no significant reduction in transfused PRBC units (I² = 84.48%). Figure 6A.

**Intra-operative Crystalloids (units)**: The MD was 0.10 (95% CI: -0.32 to 0.51, *p* = 0.64), indicating no significant difference (I² = 57.88%). Figure 6B.

**Intra-operative Colloids (units)**: The MD was − 0.25 (95% CI: -1.15 to 0.65, *p* = 0.59), showing no significant difference (I² = 92.72%). Figure 6C.

### Meta-regression

A meta-regression analysis was conducted to examine the potential moderating effects of age and sex (specifically male) on the outcomes observed in the studies. Supplementary File 1, Figs. 1–20.

- Our analysis revealed no significant association between the mean age of patients in each study and the outcomes measured, as indicated by p-values greater than 0.05 across all outcomes.
- Conversely, the proportion of male participants in each study demonstrated a statistically significant association with two specific outcomes: the length of hospitalization (days) and the difference in hemoglobin levels (g/dL). Specifically, the coefficient for the length of hospitalization was − 0.213 (p-value = 0.007), and the coefficient for the difference in hemoglobin levels was 0.245 (p-value < 0.001). For all other outcomes, no statistically significant associations were found between the number of male participants and the outcomes assessed.

### Sensitivity analysis

The sensitivity analysis revealed that excluding individual studies had minimal impact on the overall mean difference (MD) and risk ratio (RR) for most outcomes. However, two exceptions were observed. The exclusion of *Naderi et al. (2024)* [19] altered the statistical significance of the Patients’ Need for Transfusion outcome, rendering it non-significant (*p* = 0.092). Similarly, the exclusion of *Castillo-Cardiel et al. (2024)* [21] resulted in statistically significant findings in the Difference in Hemoglobin Levels (g/dL) and Differences in Hematocrit Levels (%) (*p* = 0.00 and *p* = 0.001, respectively). These results suggest that while the overall meta-analysis findings remain robust, specific studies may have a notable influence on particular outcomes. Supplementary File 2, Figs. 1, 2, 3, 4, 5 and 6.

### Cumulative analysis

The cumulative analysis provides nuanced insights into the evolution of effect sizes over time, with some outcomes demonstrating stability while others shift due to the inclusion of newer studies. The specific findings for each outcome are as follows: Supplementary File 2, Figs. 7, 8, 9, 10, 11 and 12.

- Operative Time (min): The cumulative effect size remained robust over time, with no significant deviations across studies, reinforcing the consistency of findings regarding operative duration.
- Patients’ Need for Transfusion: The addition of *Naderi et al. 2024* [19] altered the overall effect size to a statistically significant level (*p* = 0.017), suggesting that new evidence strengthens the conclusion of a reduced need for transfusion.
- Total Operative-Related Blood Loss (mL): The significance initially observed in prior studies was weakened by *Ajai et al. 2022* [22], but the overall cumulative effect size remained negative and statistically significant when later studies were added. However, the magnitude of this effect is influenced by *Naderi et al. 2024* [19] and *Castillo-Cardiel et al. 2024* [21], indicating a potential shift in the strength of the relationship.
- Presence of Post-Operative Infection: The overall effect size remained unchanged over time, suggesting a stable conclusion that surgical intervention does not significantly impact post-operative infection rates.
- Difference in Hemoglobin Levels (g/dL): The initial trend suggested a statistically significant positive mean difference based on earlier studies (*Bhatia et al. 2017* [18] and *Ajai et al. 2022* [22]). However, the inclusion of *Castillo-Cardiel et al. 2024* [21] introduced sufficient contrast or heterogeneity to neutralize this significance, leading to a final cumulative effect size that no longer supports a meaningful difference.
- Differences in Hematocrit Levels (%): A similar pattern was observed, where earlier studies (*Bhatia et al. 2017* and *Naderi et al. 2024*) indicated a significant positive effect [18, 19]. However, *Castillo-Cardiel et al. 2024* [21] contributed heterogeneity that canceled out the previously observed effect, resulting in a final cumulative effect size that lacks statistical significance.

### GRADE assessment

The GRADE assessment evaluated the certainty of evidence across four outcomes related to operative-related blood parameters. The certainty of evidence for total operative-related blood loss was rated as moderate, primarily due to concerns about the risk of bias identified in two studies. The difference in hemoglobin levels was associated with low certainty of the evidence, reflecting serious concerns regarding inconsistency across studies. Similarly, the difference in hematocrit levels was classified as having very low certainty, attributed to serious inconsistency and concerns about the risk of bias. In contrast, the certainty of evidence for patients’ need for transfusion was rated as moderate, with minor concerns regarding the risk of bias but without serious imprecision or inconsistency. Table 2.

**Table 2:** GRADE profile evidence

Certainty assessment							№ of patients		Effect		Certainty	Importance
№ of studies	Study design	Risk of bias	Inconsistency	Indirectness	Imprecision	Other considerations	TXA	Placebo	Relative (95% CI)	Absolute (95% CI)		
Total operative-related blood loss (mL)												
4	randomised trials	serious^a^	not serious^b^	not serious	not serious	none	102	102	-	MD **181.52 ml lower** (0.267 lower to 95.85 lower)	⨁⨁⨁◯ Moderate^a, b^	CRITICAL
**Difference in hemoglobin levels (gr/dL)**												
3	randomised trials	not serious	serious^c^	not serious	serious^d^	none	55	55	-	MD **0.06 gr/dL higher** (1.56 lower to 1.69 higher)	⨁⨁◯◯ Low^c, d^	IMPORTANT
**Differences in hematocrit level (%)**												
3	randomised trials	serious^a^	serious^c^	not serious	serious^d^	none	87	87	-	MD **0.19% higher** (2.85 lower to 3.23 higher)	⨁◯◯◯ Very low^a, c,d^	IMPORTANT
**Patients Need for Transfusion (%)**												
4	randomised trials	serious^a^	not serious	not serious	not serious	none	17/102 (16.7%)	45/102 (44.1%)	**RR 0.52** (0.32 to 0.85)	**212 fewer per 1**,**000** (from 300 fewer to 66 fewer)	⨁⨁⨁◯ Moderate^a^	IMPORTANT

**CI**: confidence interval; **MD**: mean difference; **RR**: risk ratio

*Explanations*

a. Two studies have an overall Some concerns risk of bias

b. Regardless I^2 = 61.46%, no individual study has a MD opposite to the overall MD

c. There was an observed statistically significant heterogeneity (I^2 > 85%)

d. Wide 95% CI

### Publication bias

Due to the limited number of included studies (five RCTs), we could not assess publication bias using Egger’s test or funnel plot visualization [16].

## Discussion

Our meta-analysis included four studies that found that IV TXA significantly reduced total operative-related blood loss and the need for transfusion compared to placebo, while Colclough et al. (2024) [20], included only in the narrative synthesis, which applied topical TXA (25 mg/mL), did not find a significant difference in bleeding parameters based on dressing weight gain, blood stain area, or visual assessment. However, this study was excluded from the meta-analysis due to differences in TXA route (topical vs. systemic) and outcome measurement (donor site bleeding vs. surgical field blood loss).

However, Colclough et al. (2024) [20] post hoc subanalysis suggested a potential benefit of TXA in cases with higher baseline bleeding, aligning with our findings on reduced blood loss. Both analyses found no significant differences in hemoglobin or hematocrit levels, operative time, length of hospitalization, and infection rates. Additionally, neither study observed a significant impact of TXA on wound re-epithelialization. While our meta-analysis found no significant difference in thromboembolic events, Colclough et al. (2024) [20] reported two cases, highlighting the need for further investigation into TXA’s safety in burn patients. The observed heterogeneity between the meta-analysis and this individual RCT may be due to the difference in the TX dosage or the application method.

### Comparison with previous Meta-Analysis

Our meta-analysis’s findings align with and expand upon previous meta-analyses while also addressing their limitations through a robust methodological approach.

#### Blood loss and transfusion requirements

These results are consistent with the findings of *Fijany et al. (2023)* [23], who also reported a significant reduction in total blood loss and PRBC transfusion in burn surgery patients receiving TXA. However, their analysis primarily included cohort studies, which are inherently more prone to confounding. In contrast, our study, which exclusively analyzed RCTs, provides more substantial evidence for the efficacy of TXA in reducing perioperative bleeding.

Similarly, *Slob et al. (2024)* [24] found moderate evidence supporting TXA’s role in reducing blood loss per unit excised area and PRBC transfusion. However, their meta-analysis failed to detect a statistically significant reduction in overall blood loss when pooling data from the two available RCTs, likely due to limited study numbers and methodological heterogeneity. Our findings build upon their work by including more RCTs, further strengthening the evidence base for TXA’s hemostatic benefits.

#### Total PRBC transfused and intraoperative fluid use

Unlike *Fijany et al. (2023)* [23], who found a significant reduction in intraoperative and total PRBC transfusions, our meta-analysis did not detect a significant difference in total PRBC units transfused. This discrepancy may stem from differences in study populations, transfusion thresholds, or surgical techniques across included trials.

Similarly, we observed no significant differences in intraoperative fluid administration between TXA and placebo, aligning with *Fijany et al. (2023)* [23], who reported no impact of TXA on intraoperative crystalloid and colloid use. These findings suggest that while TXA reduces blood loss, its effects do not necessarily translate into reduced fluid administration, possibly due to perioperative fluid management protocols varying across institutions.

#### Operative time, length of hospitalization, and postoperative infections

Our meta-analysis found no significant differences between TXA and placebo regarding operative time, hospital length of stay, and postoperative infection rates. This is consistent with *Fijany et al. (2023)* [23], who also reported no significant impact of TXA on hospital length of stay. These findings suggest that TXA effectively reduces blood loss but does not significantly alter broader surgical or recovery parameters.

Notably, our meta-regression analysis revealed that the proportion of male participants significantly influenced the length of hospitalization, suggesting that demographic factors may contribute to variability in patient recovery. This finding underscores the importance of considering patient characteristics when interpreting TXA’s effects in clinical practice.

#### Heterogeneity and weaknesses of previous studies

Our study aimed to address limitations present in previous meta-analyses. *Slob et al. (2024)* [24] included only two RCTs, leading to inconclusive findings on total blood loss. *Fijany et al. (2023)* [23] relied entirely on cohort studies prone to confounding factors. Furthermore, both meta-analyses reported significant heterogeneity, which may have been due to variations in TXA dosing, timing, and surgical techniques. Our study, by including only RCTs, minimizes some of these concerns, though heterogeneity remains an important consideration.

### Clinical relevance and implications

The significant reduction in operative blood loss (~ 180 mL) and the halving of transfusion need (RR 0.52) observed in this meta-analysis highlight clinically meaningful benefits of TXA in burn surgery. By minimizing transfusion requirements, TXA can mitigate risks such as immune reactions, infections, and fluid overload, thereby improving patient safety and outcomes. Its cost-effectiveness and widespread availability further position TXA as a practical intervention for reducing blood loss in this high-risk surgical context. However, the clinical relevance of these findings must be interpreted cautiously. While the reduction in transfusion need aligns with efforts to minimize unnecessary blood product use, the lack of significant changes in hemoglobin and hematocrit levels underscores that these biomarkers may not fully capture the clinical utility of TXA. Similarly, non-significant effects on hospital length of stay, infections, and intraoperative fluid use suggest that TXA’s benefits are likely specific to blood loss and transfusion avoidance rather than broader clinical outcomes. A key challenge lies in the variability of transfusion triggers across studies (e.g., differing hemoglobin thresholds or clinical judgment), which may explain the discrepancy between the binary outcome of “need for transfusion” and the non-significant “units of PRBC transfused.” Studies with lower transfusion thresholds may have seen larger reductions in transfusion rates even with similar absolute blood loss reductions, emphasizing the need for standardized transfusion protocols in future research.

However, our findings suggest that TXA does not significantly impact other perioperative outcomes, such as operative time, hospital length of stay, infection rates, or intraoperative fluid administration. These results indicate that while TXA effectively reduces bleeding, its effects do not extend to broader surgical or postoperative recovery parameters. This aligns with *Fijany et al. (2023)* [23], who also found no significant differences in hospital length of stay or hemoglobin and hematocrit levels.

### Safety profile of TXA in burn surgery

The included RCTs demonstrated that TXA had minimal adverse events in burn surgery. Bhatia et al. (2017) and Castillo-Cardiel et al. (2024) reported no TXA-related complications, including thromboembolic events or mortality, in either the TXA or control groups. Colclough et al. (2024) observed two isolated thrombotic events in the TXA group—a pulmonary embolism and a catheter-associated thrombus—but these did not differ statistically from the placebo group. In Naderi et al. (2024), one TXA patient experienced urticaria (a known rare side effect), which was managed appropriately, while two control-group patients with pre-existing epilepsy had postoperative seizures unrelated to TXA. No mortalities were reported across all studies.

While burn patients inherently face elevated baseline thrombotic risk due to hypercoagulability (Castillo-Cardiel et al., 2024), the included RCTs did not identify a significant increase in VTE with TXA compared to controls. This aligns with broader evidence showing TXA does not elevate thrombotic risk in surgical populations (Fijany et al., 2023). However, the small sample sizes and inconsistent monitoring of thromboembolic events across studies limit definitive conclusions.

### Value of Meta-Regression, sensitivity analysis, and GRADE in clinical application

Our meta-regression analysis provided important insights into potential sources of variability, demonstrating that the male sex significantly influenced specific outcomes, such as hospital length of stay and haemoglobin levels. This highlights the importance of considering demographic factors in future TXA studies and clinical decision-making.

Our sensitivity analysis showed that our main findings are mostly strong, but some results were unstable. For example, the significant finding that patients needed fewer blood transfusions disappeared when we excluded the Naderi et al. (2024) study. On the other hand, when we removed the Castillo-Cardiel et al. (2024) study, the previously non-significant results for hemoglobin and hematocrit changes became significant. These changes highlight how individual studies can affect overall results and remind us to interpret these outcomes carefully. The sensitivity of our findings to the inclusion of certain studies shows we should be cautious. The lack of consistency in key results, especially regarding the ‘need for transfusion,’ hemoglobin, and hematocrit changes, suggests that these effects might not be reliable in all clinical situations. This inconsistency may come from the small number of studies, small sample sizes, and large differences among patient groups, TXA dosing, and surgical methods. Therefore, the pooled effects we report for these outcomes should be seen as preliminary evidence that needs further research.

The GRADE assessment rated the certainty of evidence as moderate for total blood loss and transfusion requirements but low or very low for hemoglobin and hematocrit levels due to inconsistency and risk of bias. For clinicians, a moderate-certainty reduction in blood loss suggests that TXA can be recommended cautiously, with the understanding that future trials could refine its magnitude of benefit. A low‐certainty change in hemoglobin and a very low‐certainty change in hematocrit advise interpreting these findings with greater caution and underscore the need for larger, higher‑quality studies before drawing firm conclusions.

### Strengths of our Meta-Analysis

This systematic review and meta-analysis offers several strengths. We adhered to the PRISMA 2020 guidelines and pre-registered our protocol in PROSPERO, ensuring methodological transparency and reducing the risk of bias. Our comprehensive literature search across multiple databases, supplemented by manual reference checks, minimized the likelihood of missing relevant studies.

Additionally, we exclusively included RCTs, enhancing the reliability of our findings compared to previous meta-analyses that incorporated observational studies. Using the RoB 2 tool ensured a rigorous assessment of study quality, and our meta-regression analysis provided valuable insights into factors influencing treatment effects. Furthermore, our sensitivity and cumulative analyses strengthened the robustness of our conclusions.

### Critical methodological limitations affecting evidence synthesis

However, A notable limitation of this meta-analysis is the substantial heterogeneity observed across several key outcomes, including total operative blood loss (I² = 61.46%), hemoglobin change (I² = 91.29%), hematocrit change (I² = 88.94%), length of hospitalization (I² = 90.46%), total PRBC transfused (I² = 84.48%), and intraoperative colloid use (I² = 92.72%). This heterogeneity likely stems from clinical and methodological differences among the included randomized controlled trials. For instance, the studies varied considerably in patient populations, with inclusion criteria ranging from deep dermal thermal burns under 30% TBSA (Ajai et al., 2022) to severe burns exceeding 20% TBSA (Bhatia et al., 2017; Castillo-Cardiel et al., 2024; Naderi et al., 2024), as well as differences in burn depth, time from injury to surgery, and exclusion criteria related to comorbidities. Furthermore, the dosing and administration of TXA were heterogeneous, with intravenous doses ranging from 10 mg/kg (Naderi et al., 2024) to 15 mg/kg (Ajai et al., 2022; Bhatia et al., 2017), and one study utilizing topical TXA combined with epinephrine (Colclough et al., 2024), introducing variability in pharmacokinetics and systemic exposure. Surgical procedures also differed, including tangential excision, debridement, and split-thickness skin graft harvesting, which may influence bleeding risk and transfusion practices. Additionally, geographical and healthcare system differences across India, Norway, Mexico, and Iran could contribute to clinical management and outcomes variability. Although a random-effects model was employed to account for this diversity, the high I² values indicate that pooled estimates should be interpreted with caution, considering context (Deeks et al., 2019; Higgins et al., 2003) [25, 26]. To address the previous heterogeneity, we could not do subgroup analysis based on the route of administration or the doses due to the small number of included studies. But sensitivity analysis confirmed the robustness of our primary findings, although excluding specific studies affected the significance of some outcomes, particularly transfusion rates and hemoglobin differences. Furthermore, some studies have some concerns regarding bias overall (Bhatia et al., 2017; Naderi et al., 2024), and the sensitivity analysis played a role in addressing methodological quality and mitigating the risk of bias present in these studies. Additionally, while our meta-regression analysis identified sex as a potential moderator, other factors, such as TBSA burned, burn depth and TXA timing of administration, were not assessed due to data limitations. The few studies in this meta-analysis limited our ability to perform robust statistical assessments of publication bias. While a funnel plot was inspected, its utility was restricted by the small sample size, highlighting the need for larger studies to validate our findings. Finally, the certainty of evidence for some outcomes remained low, as reflected by GRADE assessments. The wide confidence intervals and low to very low certainty of evidence for specific outcomes highlight the risk of Type II errors (false negatives) and emphasize the need for larger-scale RCTs to confirm these results. Also, the exclusion of non-English studies, while logistically necessary, may have limited the generalizability of our findings. These limitations underscore the need for future well-designed, standardized trials with long follow-up periods to assess TXA efficacy and safety in burn surgery populations more definitively. Until then, clinicians should cautiously interpret pooled results given the underlying heterogeneity and limited statistical power, especially for secondary outcomes.

### Future research recommendations

To address the limitations of current evidence and establish a definitive risk-benefit profile for TXA in burn surgery, future research should prioritize the following:

- **Larger**,** diverse cohorts with long-term follow-up**: Well-powered, multicenter trials with predefined safety endpoints and GRADE-compliant methodologies are needed to elevate evidence certainty and confirm findings for primary and secondary outcomes. Future trials should enroll larger, heterogeneous populations to increase statistical power and reduce the risk of Type II errors (false negatives). Mortality and extended monitoring beyond the acute phase are critical to identifying rare or delayed adverse events. Burn patients face persistent thrombotic risks due to hypercoagulability, necessitating prospective evaluation of chronic outcomes and long-term safety.
- **Standardized thromboembolic surveillance**,** protocols and outcomes**: Consistent TXA dosing regimens, administration routes, and outcome definitions (e.g., transfusion triggers, burn severity metrics) are essential to minimize heterogeneity. Investigate optimal dosing regimens (e.g., intravenous vs. topical administration, timing relative to surgery) to balance efficacy and safety. Current dose variability (e.g., 10–15 mg/kg) limits study comparisons. Future trials must employ systematic protocols for VTE detection, including routine duplex ultrasonography at predefined intervals to capture both symptomatic and asymptomatic events. This will provide reliable data on TXA’s thrombotic risks in hypercoagulable burn patients.
- **Comprehensive adverse event reporting**: All potential TXA-related complications (e.g., hypotension, seizures, allergic reactions, neurological events) should be meticulously documented. Transparent reporting of patient-specific factors (e.g., pre-existing conditions like epilepsy) will refine risk-benefit assessments and clarify whether adverse events are treatment-related or attributable to baseline patient characteristics.
- **Addressing Knowledge Gaps and Clinical Caution**: Until these studies are conducted, clinicians should interpret current pooled results cautiously, particularly for secondary outcomes with low certainty of evidence (e.g., hemoglobin changes). The observed heterogeneity and statistical limitations underscore the need for individualized risk assessments in clinical practice. Subgroup analyses should evaluate TXA efficacy in patients stratified by burn severity (e.g., TBSA burned, depth), comorbidities, and surgical procedures (e.g., excision vs. grafting). This will help tailor TXA use to populations most likely to benefit.

## Conclusion

Our meta-analysis demonstrates that TXA significantly reduces total operative blood loss and transfusion requirements in burn surgery patients, aligning with previous research but offering more evidence through the exclusive inclusion of RCTs. However, the findings regarding secondary outcomes, such as operative time, hospital length of stay, postoperative infections, total PRBC transfused, and intraoperative fluid use, should be interpreted with caution due to the limited statistical power and high heterogeneity among the included studies. Clinicians should exercise caution when applying these findings to diverse clinical contexts, as the current evidence base is constrained by the limited dataset and variability in study designs. In summary, while TXA appears promising for reducing blood loss and transfusion requirements in burn surgery, further research is essential to solidify its role in clinical practice.

## Electronic supplementary material

Below is the link to the electronic supplementary material.

Supplementary Material 1

Supplementary Material 2

## Data Availability

All data generated or analyzed during this study are included in this published article [and its supplementary information files].

## Declarations

### Declaration of AI Usage

To enhance this manuscript’s clarity, readability, and academic tone, Language editing software was used for AI-assisted copy editing. This tool was employed solely to improve grammar, spelling, punctuation, and style, ensuring the text was free of linguistic errors while maintaining the integrity of the original content. The authors thoroughly reviewed and approved the final manuscript, taking full responsibility for its accuracy and originality.

## Abbreviations

TXA: Tranexamic acid
VTE: Venous thromboembolism
TBSA: Total body surface area
PRBC: Packed red blood cells
BMI: Body mass index
RCTs: Randomized controlled trials
GRADE: Grading of Recommendations Assessment, Development, and Evaluation
