# Supplementary material for: Optimizing blood management in burn surgery: a meta-analysis of tranexamic acid vs. placebo
Source: BMC Surg. 2025 Jul 11;25:294. doi: 10.1186/s12893-025-03014-4 (PMC12247390; doi:10.1186/s12893-025-03014-4)
Supplement: Supplementary file 1 — Supplementary Material 1 [file 12893_2025_3014_MOESM1_ESM.docx]

**Table 1 Search strategies**

| **Database** | **Search strategy** | **Records retrieved** |
| --- | --- | --- |
| **PubMed** | **(Tranexamic Acid OR 4-amino-methylcyclohexane-carboxyl OR 4-aminomethylcyclohexanecarbon OR 4-aminomethylcyclohexane OR 4-cyclohexane OR 4-aminomethylcyclohexanecarboxyl OR amca OR amcha OR amchafibrin OR amikapron OR aminomethyl-cyclohexane-carboxyl OR aminomethyl-cyclohexanecarboxyl OR aminomethylcyclohexane-carbon OR aminomethylcyclohexane-carboxyl OR aminomethylcyclohexanecarbon OR aminomethylcyclohexanecarboxyl OR aminomethylcyclohexanocarboxyl OR aminomethylcyclohexane OR aminomethylcyclohexano OR amstat OR anexan OR antivoff OR anvitoff OR caprilon OR cis-4-aminomethylcyclohexanecarboxyl OR cis-aminomethyl-cyclohexanecarboxyl OR cl-65336 OR cl65336 OR cyclocapron OR cyclokapron OR cyklocapron OR cyklokapron OR cyclo-F OR espercil OR exacyl OR femstrual OR fibrinon OR frenolyse OR hemostan OR hexacapron OR hexakapron OR IB-AMCA OR intrax OR kalnex OR kapron OR KABI 2161 OR lb-1148 OR lb114 OR lysteda OR micranex OR nicolda OR pause OR para-aminomethylcyclohexane-carboxyl OR rikaparin OR ronex OR rp-18429 OR rp18429 OR t-AMCHA OR Spotof OR TXA OR theranex OR transcam OR tramic OR tranex OR tranexam OR tranexan OR tranexic OR tracid OR trans-aminomethyl-cyclohexane-carboxyl OR trans-aminomethylcyclohexane-carboxyl OR trans-aminomethylcyclohexanecarboxyl OR trans-4-Aminomethylcyclohexanecarboxylic Acid OR trans-4-cyclohexanecarboxylic Acid OR transamin OR transaminomethylcyclohexane-carboxyl OR transexam OR traxamic OR trenaxin OR ugurol OR xp-12b OR xp12b) AND (burn OR burns OR burned OR burning OR escharotomy OR thermal injury OR scald)** | **685** |
| **Scopus** | **TITLE-ABS-KEY ( ( "tranexamic acid" OR txa ) AND ( "burn surgery" OR "burn injury" OR "thermal injury" ) )** | **33** |
| **Springer** | **("tranexamic acid" OR TXA) AND ("burn surgery" OR "burn injury" OR "thermal injury")** | **335** |
| **WOS** | **("tranexamic acid" OR TXA) AND ("burn surgery" OR "burn injury" OR "thermal injury") (All Fields)** | **18** |
| **Cochrane CENTRAL** | **(("tranexamic acid" OR TXA) AND ("burn surgery" OR "burn injury" OR "thermal injury")):ti,ab,k** | **16** |

**Table 2 Baseline characteristics**

| Study ID | Groups | Participant (N) | Mean age ± SD | Male, n (%) | BMI, mean ± SD | Total Body Surface Area (TBSA), mean ± SD | Burn Degree, n (%) | | | Inhalation injury, n (%) | Initial Hematocrit, mean ± SD | Initial Hemoglobin, mean ± SD |
| --- | --- | --- | --- | --- | --- | --- | --- | --- | --- | --- | --- | --- |
|  |  |  |  |  |  |  | **Superficial partial thickness** | **Deep partial thickness** | **Full thickness** |  |  |  |
| Ajai 2022 | TXA | 15 | 33.6 ± 9.51 | 12 (80) | 21.2 ± 1.7 | N/A | 0 (0) | 15 (100) | 0 (0) | N/A | N/A | 11.75 ± 1.28 |
|  | saline (placebo) | 15 | 30.7 ± 10.47 | 14 (90) | 20.9 ± 1.6 | N/A | 0 (0) | 15 (100) | 0 (0) | N/A | N/A | 12.9 ± 1.68 |
| Bhatia 2017 | TXA | 25 | 35.12 ± 8.65 | 15 (60) | 20.8 ± 1.7 | 1.7 ± 0.5 | 0 | 0 | 25(100) | N/A | 31.72 ± 2.89 | 10.34 ± 0.96 |
|  | isotonic saline (placebo) | 25 | 36.16 ± 9.22 | 14 (56) | 21.6 ± 1.9 | 1.8 ± 0.6 | 0 | 0 | 25(100) | N/A | 31.80 ± 2.77 | 10.50 ± 0.95 |
| Colclough 2024 | TXA | 23 | N/A | N/A | N/A | N/A | N/A | N/A | N/A | N/A | N/A | N/A |
|  | saline (placebo) |  |  |  |  |  |  |  |  |  |  |  |
| Naderi 2024 | TXA | 47 | 39.3 ± 13.2 | 24 (48.9) | 24.8 ± 3.5 | 45.6 ± 15.4 | 18 (45) | 18 (60) | 11 (45.8) | 7 (46.4) | 35.8 ± 4.7 | 12.5 ± 2 |
|  | saline (placebo) | 47 | 40.1 ± 12.4 | 25 (51) | 24.5 ± 3 | 43.5 ± 15.9 | 22 (55) | 12 (40) | 13 (54.1) | 8 (53.3) | 37.2 ± 4.5 | 13.2 ± 2.2 |
| Castillo-Cardiel 2024 | TXA | 15 | 33.8 ± 5.7 | 9 (60) | 27.3 ± 2.4 | 26.46 ± 5.45 | N/A | N/A | N/A | N/A | 39.6 ± 5.8 | 13.2 ± 1.7 |
|  | saline (placebo) | 15 | 35.2 ± 6.5 | 11 (73.3) | 26.2 ± 2.1 | 27.1 ± 4.45 | N/A | N/A | N/A | N/A | 38.7 ± 8.5 | 13 ± 2.3 |

# **Meta-Regression**


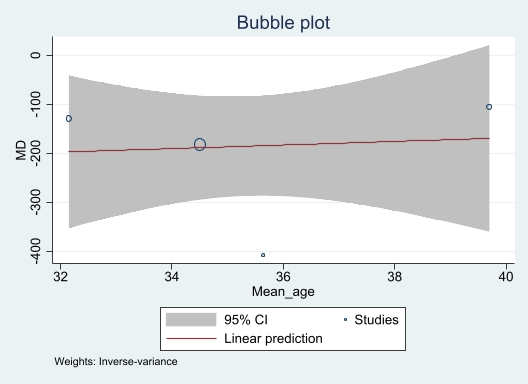


**Figure 1 Total operative-related blood loss (mL) bubble plot (age)**
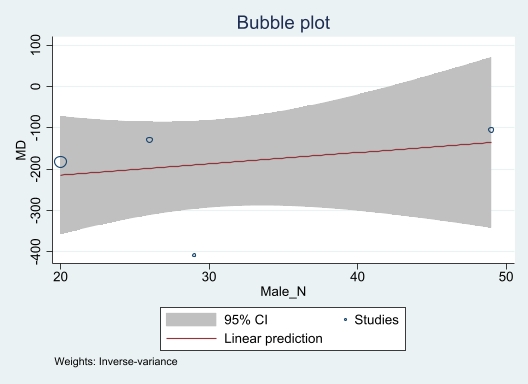


**Figure 2 Total operative-related blood loss (mL) bubble plot (sex)**


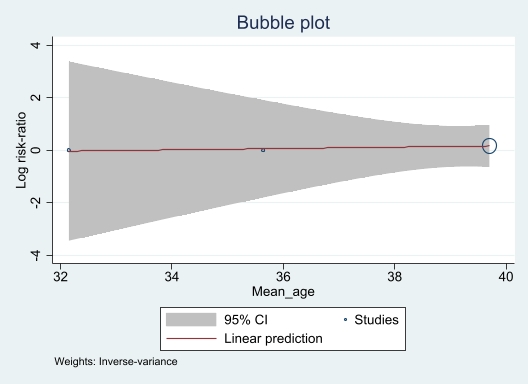


**Figure 3 Patients Need for Transfusion bubble plot (age)**


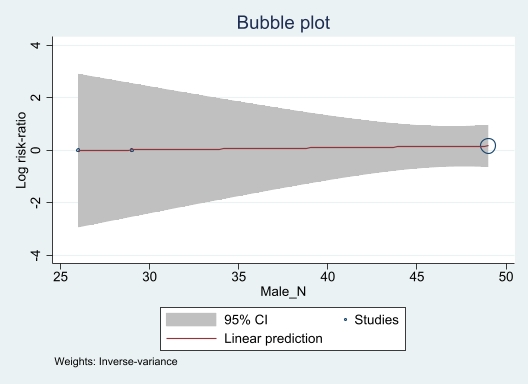


**Figure 4 Patients Need for Transfusion bubble plot (sex)**


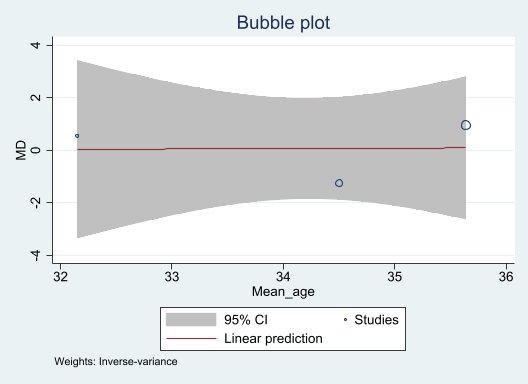


**Figure 5 Difference in hemoglobin levels bubble plot (age)**


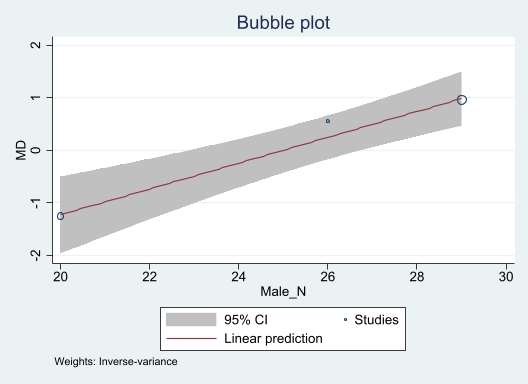


**Figure 6 Difference in hemoglobin levels bubble plot (sex)**


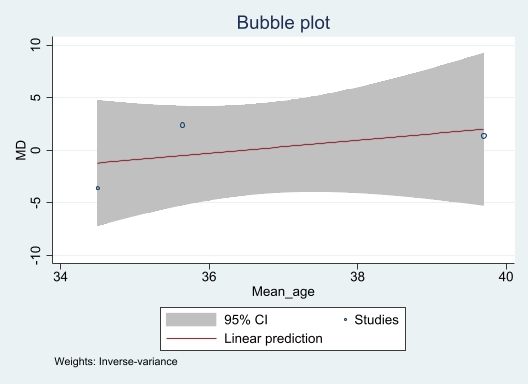


**Figure 7 Differences in hematocrit level bubble plot (age)**


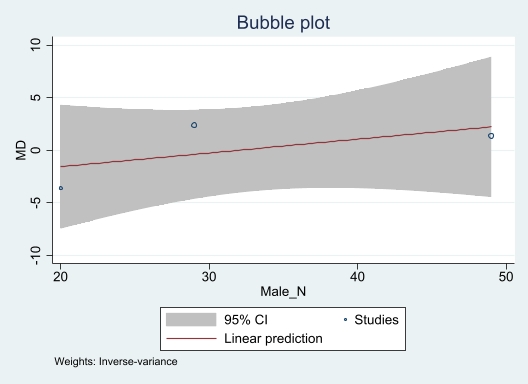


**Figure 8 Differences in hematocrit level bubble plot (sex)**


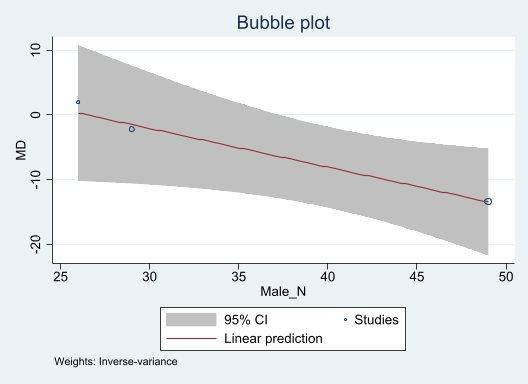


**Figure 9 Operative time (min) bubble plot (sex)**


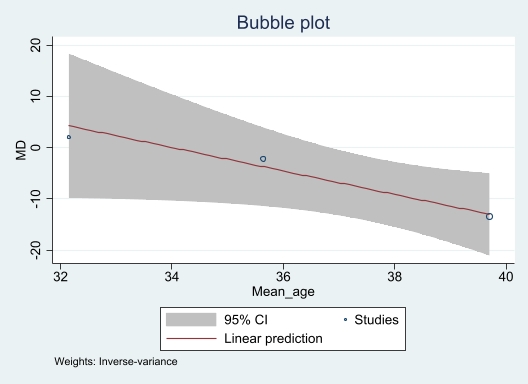


**Figure 10 Operative time (min) bubble plot (age)**


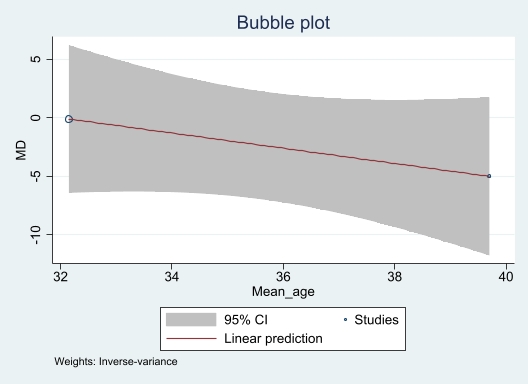


**Figure 11 Length of hospitalization (days) bubble plot (age)**


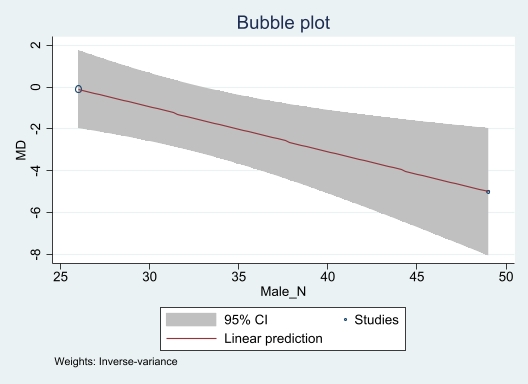


**Figure 12 Length of hospitalization (days) bubble plot (sex)**


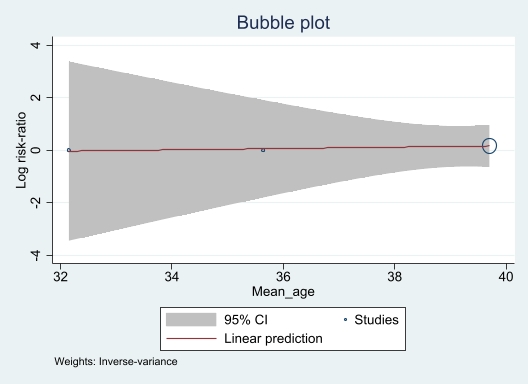


**Figure 13 Presence of post-op infection bubble plot (age)**


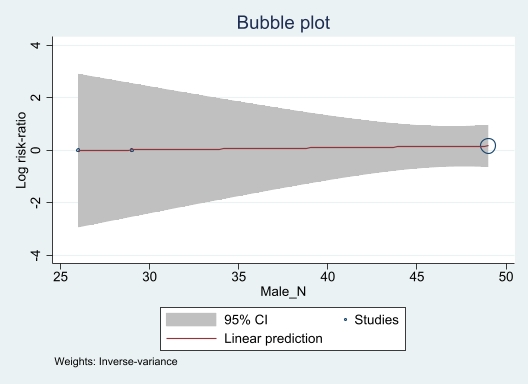


**Figure 14 Presence of post-op infection bubble plot (sex)**


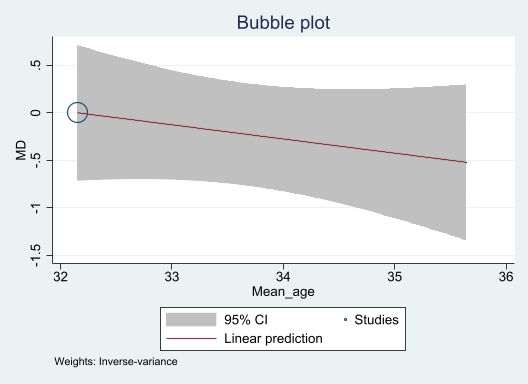


**Figure 15 Total packed red blood cells (Units) bubble plot (age)**


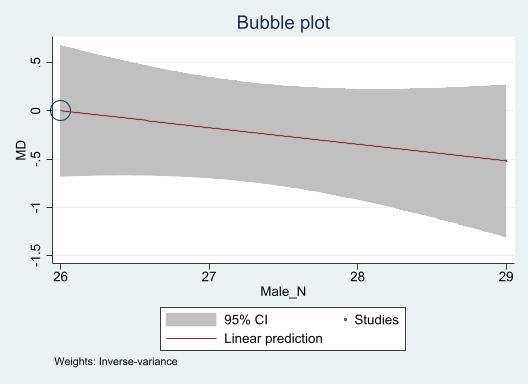


**Figure 16 Total packed red blood cells (Units) bubble plot (sex)**


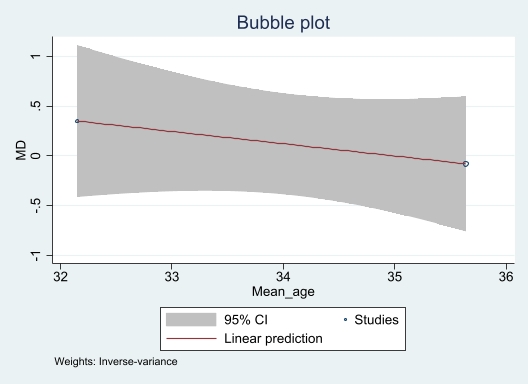


**Figure 17 Intra-operative Crystalloids (Units) bubble plot (age)**


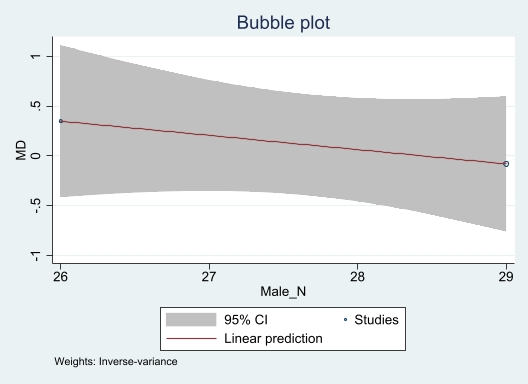


**Figure 18 Intra-operative Crystalloids (Units) bubble plot (sex)**


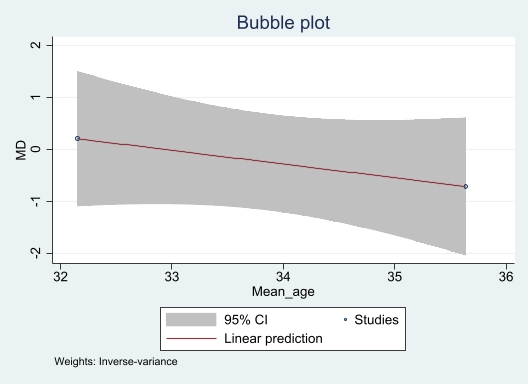


**Figure 19 Intra-operative Colloids (Units) bubble plot (age)**


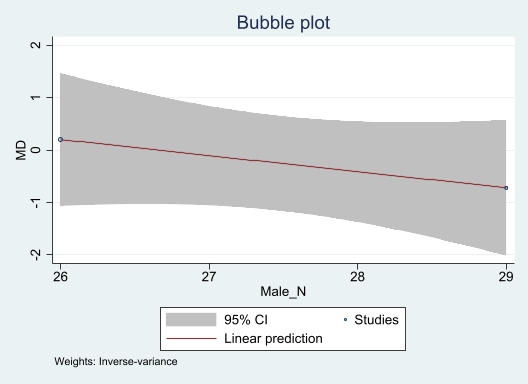


**Figure 20 Intra-operative Colloids (Units) bubble plot (sex)**
