# Supplementary material for: Optimizing blood management in burn surgery: a meta-analysis of tranexamic acid vs. placebo
Source: BMC Surg. 2025 Jul 11;25:294. doi: 10.1186/s12893-025-03014-4 (PMC12247390; doi:10.1186/s12893-025-03014-4)
Supplement: Supplementary file 2 — Supplementary Material 2 [file 12893_2025_3014_MOESM2_ESM.docx]

# **Sensitivity analysis**


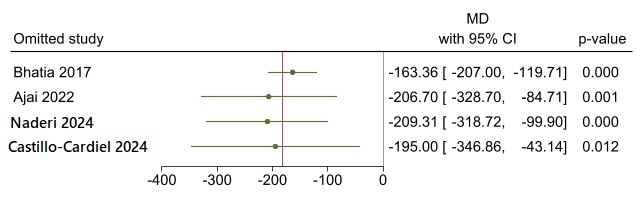


**Figure 1 Total operative-related blood loss (mL) sensitivity analysis**


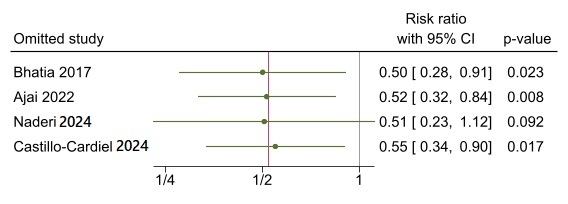


**Figure 2 Patients Need for Transfusion sensitivity analysis**


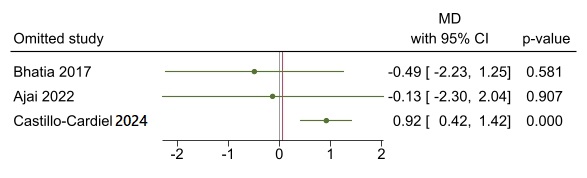


**Figure 3 Difference in hemoglobin levels sensitivity analysis**


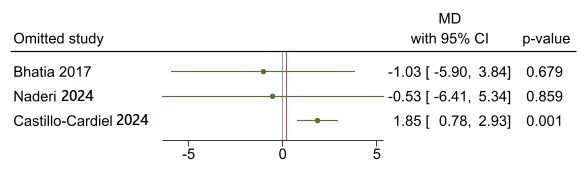


**Figure 4 Differences in hematocrit level sensitivity analysis**


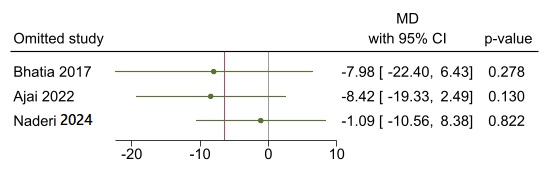


**Figure 5 Operative time (min) sensitivity analysis**


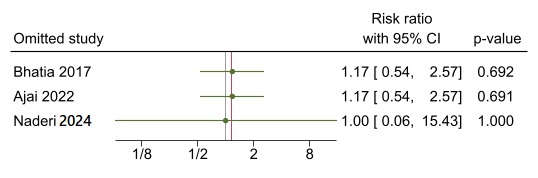


**Figure 6 Presence of post-op infection sensitivity analysis**

# **Cumulative analysis**


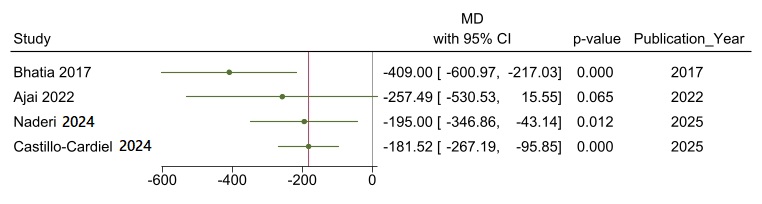


**Figure 7 Total operative-related blood loss (mL) cumulative analysis**


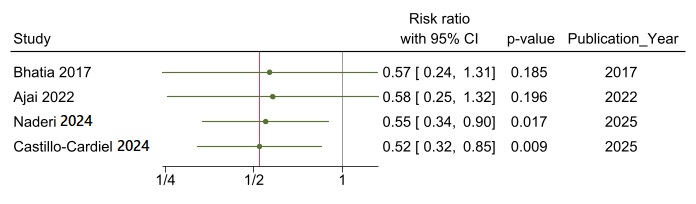


**Figure 8 Patients Need for Transfusion cumulative analysis**


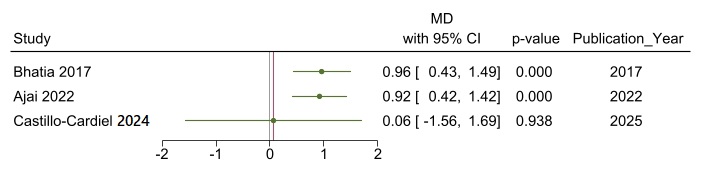


**Figure 9 Difference in hemoglobin levels cumulative analysis**


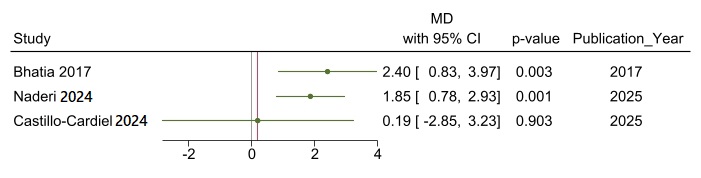


**Figure 10 Differences in hematocrit level cumulative analysis**


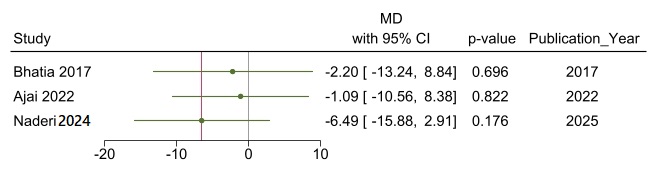


**Figure 11 Operative time (min) cumulative analysis**


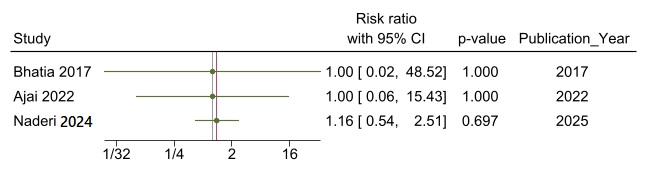


**Figure 12 Presence of post-op infection cumulative analysis**
